# Commentary: Perspective on excellence in forensic mental health services: What we can learn from oncology and other medical services

**DOI:** 10.3389/fpubh.2022.951337

**Published:** 2022-08-22

**Authors:** Maria Laura Manzone, Antonella Barbieri, Francesco Orlandini

**Affiliations:** ^1^Department of Mental Health, Psychiatric Emergency Service, Asl4 Liguria, Chiavari, Italy; ^2^Independent Researcher, Milan, Italy; ^3^Independent Researcher, London, United Kingdom; ^4^Health Care Management, Asl4 Liguria, Chiavari, Italy

**Keywords:** mental health, quality, measurement, exellence, forensic—psychiatric practice

## Introduction

Kennedy et al. have analyzed the delivery of Forensic Mental Health Services (FMHS) and focused on the quality and implementation of such Services. The Authors have observed that in other health services (i.e., Oncology) evidence-based practice is the standard routine; nevertheless, Psychiatry appears not yet to have adopted such an approach. Therefore, they have suggested that the adoption of a more evidence-based approach would allow Psychiatry to “improve treatment as usual and achieve excellence.” These inspiring conclusions have made us wonder why Psychiatry is in delay and what is the status of FMHS in Italy.

## Time and again

First, why the mission of data standardization in Psychiatry is in delayed?

According to many authors (1–3), this fact is due to several reasons.

Kennedy et al. have observed that, among all reasons, the delay is not only connected to the complexity of mental disorders but also depends on ethical concerns. Also in Italy, the debate around healthcare has often come to a standstill on philosophical topics and, more specifically, on the dichotomy between mind and brain and on biological and social factors. Dogmatic solutions and ideological issues, sometimes highly valuable, have often distracted Psychiatry from a more pragmatic and scientific approach. The anti-stigma and innovative Italian reforms are perfect examples of how the abstract discussion on philosophical topics may impact the efficacy and success of a project. In Italy, Psychiatric Hospitals were closed in 2014 and the management of healthcare was transferred from the Ministry of Justice to the Ministry of Health, with a deep cultural impact. In Italy, FMHS's pros and cons were and still are under lively debate (4) but in the end, it seems very difficult to evaluate if the Italian health facilities for forensic patients “succeed in their goals” (5) or identify the key for such evaluation. This is because the radical Italian reform of FMHS was not part of an Omni comprehensive project. As a result, it is not possible to conduct a weighted assessment of positive or negative results. The situation is different in Norway, where the massive financing has been a specific choice of political and financial programming to drive justice, correctional, and mental health programs toward a health rehabilitation approach and social inclusion.

Kennedy et al. have underlined that despite the FMHS being generally high in cost, high in risk, and low in volume services, they have a low attitude to evidence-based medicine, routine quality health assessment, and implementation. Italian FMHS can perfectly fit this description. Despite FMHS having been the target of important legislative reforms, the Italian National Health System is having great difficulties in providing information on how much is the cost of an intervention in comparison to the cost of another one and in using accountability to improve an effective service provision (6). One of the consequences that have derived from this situation is a general inefficiency that has recently brought the European Court of Human Rights to condemn Italy for the detention in an ordinary prison of an applicant suffering from psychiatric disorders, despite a domestic court had ordered his transfer to a residential center (7).

Substantial improvements can be surely achieved in the short term, but we struggle to believe that the “precision medicine” mentioned by Kennedy et al. can be a near and imminent goal for Psychiatry as it is for other disciplines, such as Oncology, among others. The innovations that have changed the mental health care field have in fact benefited less than other medical disciplines from the technological revolution of recent years. But now “big data are coming to Psychiatry” (8), so we can be optimistic for at least the medium and long term. The ambitious international projects, financed with a number of funds never seen before, in the public-private partnership and which involved multiple participants and affiliates, trigger enthusiasm, make us feel the development's fast pace in this field and the hope to be part of it. The Brain initiative, the European Brain Project, and the accelerating Medicines Partnership for Schizophrenia recently announced by the National Institute of Mental Health are just some examples. Responsible Neuroscience shows us the path to follow to understand “how social science, ethics, and philosophy can become driving forces in scientific and technological development” (9). In 6 years, NIMH has awarded more than 400 grants for technology-enhanced mental health interventions designed to prevent or treat mental disorders. “Technology has opened a new frontier in mental health support and data collection” (NIMH website).

For all these reasons, we share the opinion of Kennedy et al.', who say “there is no reason why this should not now be achievable.”

We see no obstacles to increased use of technology in mental health support: “the healthcare sector will face the challenges and opportunities presented by the volume, variety, and complexity of big data” (10), like any other area of science and services. We want to underline that some of the more prestigious epidemiologic research on mental health, analysis of high volume and high variety of data, have focused on detainee patients, an area of forensic psychiatry. The output of such research has been the driver for the Institutions to move forward and adopt innovative best practices, draw the pathway for the lawmakers, and, even more difficult to achieve, induce a diffuse cultural change (11–13).

“Clinical trials are better, faster, cheaper with big data” (14) and more ethical in some cases. Psychiatry and FMHS are a part of Psychiatry and are facing big data advances (15–17). The processing of a vast amount of data makes it more and more difficult to observe what happens inside the black box of big data. For Psychiatry, the diagnosis continues to be clinical diagnosis and it is a giant leap to place our trust in data-driven research instead than in hypothesis-driven research (8, 18). But psychiatrists should not lose the ability to think critically. There are some abilities in psychiatric practice that make it easier to remain human-centered: the attitude to create a relationship with the patient and the caregiver, taking into consideration multiple determinants of health, as well as connecting treatment and care. Big data in mental health are a bridge between past and future ethics.

## Discussion

As clinicians, we have appreciated the Kennedy et al. article, that proposes quality standards, excellence, and effectiveness with great respect for the wide heterogeneity of the FMHS and for the clinical practice we are dealing with daily. Measurements must be applied to impact patient safety and quality of healthcare (4, 16, 17, 19, 20). The Authors proposed a hierarchical model for levels and domains. This hierarchical model mirrors the usual clinical and legal decision-making we are used to. By doing so, Kennedy et al. encourage us to continue in a framework to “improve treatment as usual and achieve excellence.”

## Author contributions

All authors listed have made a substantial, direct, and intellectual contribution to the work and approved it for publication.

## Conflict of interest

The authors declare that the research was conducted in the absence of any commercial or financial relationships that could be construed as a potential conflict of interest.

## Publisher's note

All claims expressed in this article are solely those of the authors and do not necessarily represent those of their affiliated organizations, or those of the publisher, the editors and the reviewers. Any product that may be evaluated in this article, or claim that may be made by its manufacturer, is not guaranteed or endorsed by the publisher.

## Author disclaimer

The views expressed in the article are those of the Authors.
